# Moral Distress and Turnover Intention Among Nurses in Saudi Arabia: A Cross-Sectional Study

**DOI:** 10.3390/healthcare14213669

**Published:** 2026-10-28

**Authors:** Ali Alharbi, Essa Hakamy

**Affiliations:** 1 Medical Surgical Department, College of Nursing, King Saud University, Riyadh P.O. Box 642, Saudi Arabia; 2 Department of Nursing Administration and Education, College of Nursing, King Saud University, Riyadh P.O. Box 642, Saudi Arabia; ehakamy@ksu.edu.sa

**Keywords:** moral distress, turnover intention, intensive care unit, oncology, emergency department, Saudi Arabia

## Abstract

**Highlights:**

**What are the main findings?**

- Among 160 nurses working in intensive care, oncology, and emergency departments in Saudi Arabia, higher moral distress was associated with greater turnover intention.
- Moral distress differed significantly across clinical departments, with intensive care and oncology nurses reporting higher levels than emergency department nurses.

**What are the implications of the main findings?**

- These findings support recognizing and addressing moral distress as an important component of workforce retention strategies in high-acuity nursing settings.
- Organizational approaches that strengthen ethical support, supportive leadership, and opportunities for nurses to address ethically challenging situations may help reduce moral distress and promote workforce retention.

**Abstract:**

Background/Objectives: Moral distress is a common ethical challenge among nurses working in high-acuity clinical settings and is often associated with turnover intention. However, evidence regarding this relationship among nurses in Saudi Arabia remains limited. This study examined the relationship between moral distress and turnover intention to identify factors associated with turnover intention among nurses. Methods: A cross-sectional study was conducted among 160 registered nurses working in intensive care, oncology, and emergency departments in Saudi Arabia. Three instruments were used for data collection: a sociodemographic questionnaire developed by the research team, the Measure of Moral Distress for Healthcare Professionals, and the Turnover Intention Scale. Results: Nurses working in intensive care and oncology units reported significantly higher moral distress than those working in emergency departments (*F* = 5.979, *p* = 0.003). Female nurses reported significantly higher moral distress than male nurses (*t* = 1.980, *p* = 0.049). Pearson’s correlation analysis revealed a significant positive association between moral distress and turnover intention (*r* = 0.332, *p* < 0.001). Multiple linear regression analysis, controlling for sex, nationality, educational level, department, and years of clinical experience, revealed that moral distress remained significantly associated with turnover intention (β = 0.367, *p* < 0.001); the model explained 15.0% of the variance in turnover intention (R^2^ = 0.150; adjusted R^2^ = 0.116). Conclusions: Nurses experiencing higher levels of moral distress reported greater turnover intention, and this association remained significant after adjustment for selected sociodemographic characteristics. Healthcare organizations may consider strategies to address moral distress, such as fostering supportive ethical work environments, strengthening ethical leadership, and promoting nurses’ well-being, to support workforce retention. However, the cross-sectional design and convenience sampling limit causal inference and the generalisability of these findings.

## 1. Introduction

In acute care, moral distress is a widespread concern for the sustainability of the nursing profession. Jameton’s original concept of moral distress suggested that a range of institutional, hierarchical, and contextual factors may restrain nurses from performing a course of action that they believe is morally right [1,2]. Unlike emotional stress, which is associated with failure to cope, moral distress is associated with ethical conflict, and its psychological effects are more enduring, thus affecting nurses’ physical and psychological well-being and the quality of patient care [2]. Turnover intention, which in this case refers to a nurse’s deliberate plan to leave a position, unit, or profession, is a critical issue for workforce stability [3].

High-acuity areas such as intensive care units (ICUs), emergency departments (EDs), and oncology units are disproportionately affected. According to a meta-analysis, the turnover intention rate among ICU nurses was 27.7%, significantly higher than that in other departments [2]. Up to 51% of ED nurses stated that they had left or were thinking of leaving their positions, explicitly mentioning moral distress as a reason [4]. Compared with critical care nurses, oncology nurses have lower levels of moral distress, although they have similar trends in burnout and plans to leave, with burnout related to extended end-of-life conflicts, nonbeneficial treatments, and barriers to providing compassionate care [5]. These patterns are similar across international contexts.

A systematic review and meta-analysis of 86 studies involving 19,537 participants from 21 countries revealed a pooled moral distress score of 2.55 out of 10, with a much higher score in developing countries [6]. Ahmad et al. [3] found that 71.9% of ICU nurses in the United Arab Emirates (UAE) experienced severe moral distress, which was associated with 3.73 times the odds of planning to leave the profession compared with mild moral distress. In China, a study of emergency nurses showed that hospital ethical climate and moral resilience mediated the relationship between moral distress and job burnout, suggesting that the organizational environment is closely linked to nurses’ job outcomes [7]. Burnout was found to significantly mediate the relationship between moral distress and turnover intention among Korean nurses during the COVID-19 pandemic, suggesting that unresolved moral distress may gradually lead to nurse turnover [8].

### 1.1. Rationale and Literature Gap

Although it is well established internationally that moral distress is associated with turnover intention, evidence regarding this relationship in Saudi Arabia remains limited. A systematic review of 21 studies identified work environment and organizational factors as being associated with nursing turnover in Saudi Arabia; however, it did not identify moral distress as a distinct construct [9]. A national cross-sectional study revealed that 53.7% of Saudi nurses intended to leave their jobs; the proportion was highest in critical care, and lower salary was the factor most strongly associated with this intention. However, moral distress was not assessed [10]. This represents an important gap in the literature, as Saudi Arabia has a hierarchical healthcare system with a structure similar to that of the UAE, where a strong association between moral distress and turnover intention has been observed [3].

Additionally, evidence from multispecialty comparisons across ICU, ED, and oncology settings in Saudi Arabia is limited. International evidence demonstrates variations in the sources of moral distress by specialty. Although these populations are usually studied separately, the ethical challenges of each are important: futile life-sustaining interventions in ICUs, rapid ethical decision-making in EDs, and the conflict between therapeutic benefit and compassionate care in oncology units. Furthermore, in ICU settings, ethical climate has been reported to moderate the relationship between moral distress and turnover intention [11], which has not been explored in Saudi Arabia. Filling these gaps will generate contextually relevant evidence to support retention policy development.

### 1.2. Purpose and Significance of the Study

This study aimed to examine the association between moral distress and turnover intention among nurses working in ICUs, oncology units, and EDs in Saudi Arabia. Specifically, it aimed to (1) describe the sociodemographic characteristics and factors associated with moral distress and turnover intention among nurses working in ICUs, oncology units, and EDs; (2) examine the relationships among selected sociodemographic characteristics, moral distress, and turnover intention in these nurses; and (3) examine the variance in turnover intention explained by selected sociodemographic characteristics and moral distress. The study provides a comparative examination of moral distress and turnover intention among nurses across multiple high-acuity specialties in Saudi Arabia.

This study is significant from clinical, organizational, and policy perspectives. For bedside nurses, identifying the triggers of moral distress can inform focused ethical support and mental health resources. For healthcare organizations, the findings may inform investment in technology and resources for assessing the ethical climate and for structured debriefing, along with the use of shared decision-making processes, which have been associated with lower distress and higher retention [3,7]. The findings may help policymakers decide how best to guide the development and retention of the nursing workforce in accordance with the Saudi Vision 2030 policies for the health system. For researchers, this study extends the current knowledge from the Gulf region and lays the groundwork for future research in these areas.

### 1.3. Theoretical Framework

This study is based on two theoretical frameworks. The first is Jameton’s original conceptualization of moral distress, which posits that if moral distress remains unresolved within a nurse over time, the nurse begins to accumulate “moral residue,” which threatens the nurse’s moral integrity until they reach a point of frustration and resignation [1,12]. If such a state of moral distress remains unresolved, it may contribute to nurse burnout and turnover intention. The second theoretical framework is Hobfoll’s conservation of resources (COR) theory, which suggests that stress is experienced when individuals’ personal and professional resources are threatened or lost or become unavailable despite their significant investments [13]. Although organizations may work to replenish these resources, resource depletion may continue among the nurses themselves, which may lead to outcomes such as turnover intention [7]. In the present study, Jameton’s conceptualization of moral distress and COR theory were used as conceptual lenses for understanding the association between moral distress and turnover intention.

## 2. Materials and Methods

### 2.1. Study Sample and Design

This study used a descriptive cross-sectional design. The design was considered appropriate because it enables the examination of associations among variables within a defined population at a single point in time and is widely used in observational nursing research [14,15].

The study was conducted in hospitals across Saudi Arabia that provide intensive care, oncology, and emergency services. It included hospitals from all regions of Saudi Arabia to ensure broad geographical representation across the Kingdom. Nurses were recruited using non-probability convenience sampling.

The required sample size was estimated using G*Power version 3.1.9.6 for multiple linear regression analysis [16]. The test family selected was F-tests, using the ‘Linear Multiple Regression: Fixed model, R^2^ deviation from zero’ procedure, which evaluates the significance of the overall regression model. Assuming a medium effect size (Cohen’s *f*^2^ = 0.15), a statistical power of 80%, a significance level of 0.05, and 6 predictors, the minimum required sample size was 103 participants. To compensate for potential incomplete responses, the target sample size was increased by approximately 10% to 113 participants. A total of 160 nurses completed the survey, exceeding the minimum sample size estimated by the a priori power analysis [16,17].

Eligible participants were registered nurses currently working in ICUs, oncology units, or EDs who had at least six months of clinical experience in their current unit and were able to read and understand English. Nurses working exclusively in administrative or managerial positions, nursing interns, and those on extended leave during the data collection period were excluded.

Participation was voluntary, and electronic informed consent was obtained from all participants before they accessed the questionnaire. Participants were assured that their responses would remain anonymous and confidential and that they could withdraw from the study at any stage without consequence. Ethical approval was obtained from the Institutional Review Board of King Saud University (Approval No. KSU-HE-25-1405), which was deemed sufficient for data collection across all participating institutions.

### 2.2. Data Collection

Data were collected between December 2025 and June 2026 using an online self-administered questionnaire developed and distributed via the Qualtrics platform, which enabled secure, anonymous, and geographically flexible data collection across healthcare institutions in Saudi Arabia. The online survey was reported in accordance with the Checklist for Reporting Results of Internet E-Surveys (CHERRIES) [18]. The survey link was disseminated directly to eligible nurses working in ICUs, oncology units, and EDs. These specialties were selected as the predefined clinical settings of interest for this study and were not intended to represent all high-acuity nursing environments. Other relevant settings, including operating rooms, trauma units, and burn units, were outside the predefined scope of the study. The survey was distributed broadly through professional nursing networks across all regions of Saudi Arabia. As the questionnaire was distributed via an open online link, the exact number of nurses approached could not be determined; therefore, a formal response rate could not be calculated. To ensure data quality, only fully completed questionnaires were included in the analysis. The Qualtrics platform was configured to require responses to all items before submission, thereby minimizing the occurrence of incomplete responses. At the outset of the survey, participants were provided with an information sheet outlining the study purpose, the confidentiality of their responses, and their right to withdraw at any time without consequence. Informed consent was obtained electronically prior to participation. To ensure anonymity, no personally identifying information was collected, and all responses were used solely for research purposes.

### 2.3. Variables and Instruments

#### 2.3.1. Sociodemographic Characteristics

The research team developed a sociodemographic questionnaire to capture key participant characteristics relevant to the study objectives. The instrument comprised six items assessing age, sex, nationality, years of clinical experience, highest educational level attained, and current clinical department. These variables were selected based on their established relevance in the nursing workforce literature and their potential to explain variations in moral distress and turnover intention [6,9].

#### 2.3.2. Moral Distress

Moral distress was measured using the Measure of Moral Distress for Healthcare Professionals (MMD-HP), developed by Epstein et al. [19] to assess the frequency and intensity of morally distressing situations experienced by healthcare professionals. The instrument comprises 27 items representing ethically challenging situations commonly encountered in clinical practice. Each item is evaluated using two 5-point Likert scales: one measuring the frequency of exposure to the situation, ranging from 0 (never) to 4 (very frequently), and the other measuring the level of distress caused by the situation, ranging from 0 (none) to 4 (very distressing). Following the recommended scoring procedure, the frequency and distress scores for each item are multiplied to generate a composite item score (frequency × distress) ranging from 0 to 16, and the total moral distress score is calculated by summing all 27 composite item scores, resulting in a possible total score ranging from 0 to 432. Higher scores indicate greater levels of moral distress. The MMD-HP has demonstrated excellent psychometric properties across diverse healthcare settings, with reported Cronbach’s alpha coefficients exceeding 0.90 [19]. In the present study, the instrument demonstrated excellent internal consistency, with a Cronbach’s alpha coefficient of 0.974.

#### 2.3.3. Turnover Intention

Turnover intention was measured using the three-item Turnover Intention Scale (TIS), originally developed by Michaels and Spector [20]. Each item is rated on a 6-point Likert scale ranging from 1 (strongly disagree) to 6 (strongly agree). A mean turnover intention score was calculated for each participant by averaging the responses to the 3 items, resulting in a possible score range of 1 to 6, with higher scores indicating greater turnover intention. The TIS has been widely used in nursing and healthcare research internationally and has demonstrated acceptable to good internal consistency, with Cronbach’s alpha values typically ranging from 0.77 to 0.89 in nursing samples [21,22]. The Cronbach’s alpha for the current study was 0.908, indicating excellent internal consistency.

### 2.4. Data Analysis

Data were analyzed using the Statistical Package for the Social Sciences (SPSS) version 29. Descriptive statistics were used to summarize sample characteristics and study variables, with continuous variables presented as means (M) and standard deviations (SD) and categorical variables reported as frequencies and percentages. Composite scores for the MMD-HP were computed by multiplying the frequency and distress scores for each of the 27 items and summing the resulting products, in accordance with the scoring procedure recommended by Epstein et al. [19]. The TIS score was computed as the mean of its three items, with higher scores indicating greater turnover intention.

Inferential statistical analyses were conducted in accordance with the study objectives. Independent samples t-tests and one-way analysis of variance (ANOVA) were used to examine mean differences in moral distress and turnover intention across sociodemographic subgroups. Where significant ANOVA results were identified, Tukey’s post hoc test was applied to determine specific between-group differences. Pearson’s correlation coefficient (*r*) was used to assess the relationships between continuous variables, including age, years of clinical experience, moral distress, and turnover intention. The strength of correlation coefficients was interpreted according to Cohen [23], whereby values of 0.10, 0.30, and 0.50 denote small, medium, and large effects, respectively. Multiple linear regression analysis using the Enter method was conducted to examine the association of moral distress and selected sociodemographic variables with turnover intention. The variables entered into the regression model were sex, nationality, educational level, department, years of clinical experience, and total moral distress score. Sex was coded 0 = male, 1 = female; nationality 1 = Saudi, 2 = expatriate; educational level 1 = undergraduate, 2 = postgraduate; and department 1 = ICU, 2 = oncology, 3 = ED. The ordinal treatment of department is acknowledged as a limitation, given that department did not reach statistical significance (*p* = 0.071). Multicollinearity was assessed using tolerance and variance inflation factor (VIF) statistics; all independent variables in the final model returned tolerance values above 0.10 and VIF values below 10, confirming the absence of problematic multicollinearity. A significance level of *p* < 0.05 was applied for all analyses. Standard regression assumptions were examined before analysis, including linearity, normality of residuals, homoscedasticity, and independence of errors, through standard SPSS diagnostic plots; no substantial violations were identified.

## 3. Results

### 3.1. Sociodemographic Characteristics of the Study Sample

A total of 160 registered nurses working in ICU, oncology, or ED settings across Saudi Arabia participated in this study. The sociodemographic characteristics of the participants are presented in Table 1. Most participants were female (62.5%). Regarding nationality, expatriate nurses represented a slightly larger proportion of the sample (53.8%) than Saudi nurses (46.3%). In terms of educational background, most participants (85.0%) held an undergraduate degree as their highest educational level. The largest proportion of nurses worked in ICUs (43.8%), followed by oncology units (32.5%) and EDs (23.8%). The participants had a mean age of 33.37 years (SD = 7.23), with ages ranging from 22 to 60 years. Their mean clinical experience was 8.50 years (SD = 6.11), ranging from less than one year to 27 years.

### 3.2. Levels of Moral Distress and Turnover Intention Among ICU, Oncology, and ED Nurses

Table 2 presents the descriptive statistics and internal consistency estimates for the MMD-HP and the TIS.

The mean total moral distress score was 128.89 (SD = 103.28), with scores ranging from 0 to 404 out of a possible maximum of 432, indicating some level of moral distress within the sample. The MMD-HP demonstrated excellent internal consistency (Cronbach’s α = 0.974).

At the item level, the MMD-HP composite scores (frequency × distress) for each of the 27 items ranged from 0 to 16. Item 19 “*Having excessive documentation requirements that compromise patient care*” had the highest composite score (M = 6.03, SD = 5.61), followed by item 3 “*Feeling pressured to order or carry out orders for what I consider to be unnecessary or inappropriate tests and treatments*” (M = 5.92, SD = 5.28) and item 16 “*Being required to care for more patients than I can safely care for*” (M = 5.44, SD = 5.08). By contrast, item 21 “*Feeling unsafe or bullied amongst colleagues*” had the lowest composite score (M = 3.98, SD = 4.62).

Regarding the frequency dimension of moral distress, nurses most frequently encountered item 2 “*Following the family’s insistence to continue aggressive treatment even though it is not in the best interest of the patient*” (M = 2.29, SD = 1.36), followed by item 3 “*Feeling pressured to order or carry out orders for unnecessary or inappropriate tests and treatments*” (M = 2.16, SD = 1.28) and item 19 “*Having excessive documentation requirements that compromise patient care*” (M = 2.16, SD = 1.30). Item 15 “*Feeling pressured to ignore situations in which patients have not been given adequate information to ensure informed consent*” was the least frequently experienced situation (M = 1.62, SD = 1.39).

Regarding the level of distress, item 3 “*Feeling pressured to order or carry out orders for unnecessary or inappropriate tests and treatments*” was the most distressing situation (M = 2.21, SD = 1.37), followed by item 16 “*Being required to care for more patients than I can safely care for*” (M = 2.18, SD = 1.34) and item 5 “*Continuing to provide aggressive treatment for a person who is most likely to die when no one will make a decision to withdraw it*” (M = 2.15, SD = 1.29). Item 11 “*Witnessing a violation of a standard of practice or code of ethics*” was the least distressing situation (M = 1.76, SD = 1.46).

Regarding turnover intention, the mean score on the TIS was 2.83 (SD = 1.19) out of a possible maximum of 6, with higher scores indicating greater turnover intention. The TIS demonstrated excellent internal consistency (Cronbach’s α = 0.908). The highest mean score was recorded for item 3 “*I have started to look for other jobs*” (M = 2.92, SD = 1.47), followed by item 1 “*I often seriously consider leaving my current job*” (M = 2.82, SD = 1.20). Item 2 “*I intend to quit my current job*” had the lowest mean score (M = 2.75, SD = 1.21).

### 3.3. Factors Associated with Moral Distress and Turnover Intention

Table 3 presents the associations between participants’ sociodemographic characteristics and both moral distress and turnover intention. Neither age nor years of clinical experience was significantly associated with moral distress or turnover intention (*p* = 0.074 and *p* = 0.147, respectively) or turnover intention (*p* = 0.711 and *p* = 0.245, respectively). Similarly, nationality and educational level were not significantly associated with either outcome.

Female nurses reported significantly higher moral distress scores than male nurses (140.16 ± 115.51 vs. 109.98 ± 75.84, *t* = 1.980, *p* = 0.049, Cohen’s d = 0.294, 95% CI for mean difference [0.07, 60.28]), whereas turnover intention did not differ significantly between sexes (*p* = 0.950). Although the difference reached the conventional threshold for statistical significance, the marginal *p*-value and the number of subgroup comparisons warrant a cautious interpretation of this finding.

Clinical department was significantly associated with both moral distress (*F* = 5.979, *p* = 0.003) and turnover intention (*F* = 3.125, *p* = 0.047). Nurses working in ICUs reported the highest moral distress scores, followed by oncology nurses, whereas ED nurses reported the lowest scores. Tukey’s post hoc analysis demonstrated significantly higher moral distress among ICU nurses than among ED nurses (*p* = 0.002) and among oncology nurses than among ED nurses (*p* = 0.037). No significant difference was observed between ICU and oncology nurses. The departmental comparison yielded an eta-squared effect size of 0.072 (95% CI [0.009, 0.153]), indicating that clinical department accounted for approximately 7.2% of the observed variance in moral distress. Although the overall ANOVA indicated a significant difference in turnover intention across departments (F = 3.125, *p* = 0.047), Tukey’s post hoc comparisons did not identify significant pairwise differences between ICU and oncology (adjusted *p* = 0.056, 95% CI [−1.010, 0.010]), ICU and ED (adjusted *p* = 0.999, 95% CI [−0.570, 0.553]), or oncology and ED (adjusted *p* = 0.127, 95% CI [−0.104, 1.086]).

### 3.4. Relationship Between Moral Distress and Turnover Intention

Pearson’s correlation analysis was conducted to examine the relationship between moral distress and turnover intention. The results revealed a statistically significant positive correlation between moral distress and turnover intention (*r* = 0.332, *p* < 0.001), indicating that nurses with higher levels of moral distress reported greater turnover intention (Table 4).

### 3.5. Multiple Linear Regression Analysis of Turnover Intention Among ICU, Oncology, and ED Nurses

A multiple linear regression analysis was conducted to examine whether selected sociodemographic characteristics and moral distress were associated with nurses’ turnover intention. The final model included sex, nationality, years of clinical experience, educational level, department, and moral distress as independent variables (Table 5).

The regression model was statistically significant (*F*(6, 153) = 4.433, *p* < 0.001), explaining 15.0% of the variance in turnover intention (R^2^ = 0.150; adjusted R^2^ = 0.116). The remaining 85% of variance is attributable to factors not captured in the present model.

Moral distress was independently associated with turnover intention after adjustment for selected covariates (B = 0.004, β = 0.367, *t* = 4.652, *p* < 0.001). To contextualise this effect, a 100-point increase in MMD-HP score was associated with an estimated 0.40-point increase in turnover intention on the six-point scale, indicating a statistically significant but modest association. This finding indicates that higher levels of moral distress were associated with greater turnover intention among nurses.

Sex, nationality, years of clinical experience, educational level, and department were not significantly associated with turnover intention after adjustment (all *p* > 0.05); even the lowest *p* values, those for nationality (*p* = 0.082) and department (*p* = 0.071), did not reach statistical significance.

## 4. Discussion

This study examined the relationship between moral distress and turnover intention among nurses working in ICUs, oncology units, and EDs in Saudi Arabia. Overall, nurses reported some level of moral distress and turnover intention. Moral distress differed significantly according to sex and clinical department, whereas turnover intention varied only across clinical departments. A significant positive correlation was observed between moral distress and turnover intention, indicating that nurses with higher levels of moral distress reported greater turnover intention. Furthermore, after adjustment for the included sociodemographic characteristics, moral distress was the only variable significantly associated with turnover intention in the regression model. However, the overall model accounted for 15% of the variance in turnover intention, suggesting that moral distress may be one of several organizational and professional issues related to workforce stability in high-acuity nursing settings.

The observed level of moral distress is consistent with international evidence demonstrating that ethically challenging situations remain an inherent aspect of nursing practice in critical care environments. Alimoradi et al. [6] reported that moral distress is a widespread phenomenon among nurses worldwide, particularly in demanding clinical settings, and Ahmad et al. [3] identified high levels of moral distress among ICU nurses in the UAE. Although the level of moral distress observed in the present study was lower than that reported in the UAE, both studies highlight the persistent ethical challenges encountered by nurses caring for critically ill patients. This difference may reflect variations in organizational culture, ethical support mechanisms, staffing resources, or healthcare policies between the two healthcare systems rather than differences in the ethical demands of nursing practice itself. At the item level, the situations with the highest composite moral distress scores were predominantly organizational and systemic, including excessive documentation requirements, pressure to carry out unnecessary or inappropriate clinical interventions, and insufficient staffing levels relative to patient acuity. These findings indicate that institutional constraints were among the prominent sources of moral distress reported by participants in this sample. This is theoretically consistent with Hobfoll’s COR theory [13], which posits that occupational stress arises when valued professional resources, including time, autonomy, and the capacity to deliver safe and ethical care, are threatened or depleted by institutional demands. From a Jametonian perspective, these constraints represent the external barriers that prevent nurses from acting in accordance with their moral judgment and may contribute to the psychological suffering that is characteristic of moral distress [1].

The level of turnover intention observed in this study is consistent with growing international concerns regarding workforce retention in high-acuity nursing environments. Previous studies conducted in Saudi Arabia have primarily attributed nurses’ turnover intention to organizational factors such as workload, salary, leadership, and work environment [9,10]. The present findings broaden this understanding by suggesting that turnover intention may also be viewed from an ethical perspective. Specifically, the findings suggest that nurses’ intentions to remain in or leave their positions may be related to both organizational conditions and repeated exposure to ethically distressing situations during clinical practice. This adds to the Saudi nursing literature, where moral distress has received relatively limited attention as a factor potentially associated with workforce retention. It should be noted that turnover intention, as measured in this study, reflects nurses’ self-reported cognitive inclination to leave their current position and does not necessarily predict actual resignation or departure.

Moral distress was independently associated with turnover intention after adjustment for selected sociodemographic characteristics. Although the bivariate correlation was of medium strength, moral distress remained associated with turnover intention in the regression model, whereas none of the sociodemographic characteristics in the model (sex, nationality, years of clinical experience, educational level, and department) was significantly associated with turnover intention after adjustment. These findings indicate that moral distress was associated with turnover intention independently of the sociodemographic characteristics included in the model; however, its relative contribution compared with unmeasured organisational and work-related factors remains uncertain. This finding extends the existing evidence by showing that moral distress is independently associated with turnover intention within the Saudi healthcare context. Nevertheless, a reverse or reciprocal relationship cannot be excluded, as nurses already contemplating leaving their employment may perceive or report ethically challenging workplace situations differently, potentially influencing their reported levels of moral distress. Previous studies have consistently reported positive associations between moral distress and adverse workforce outcomes, including burnout, job dissatisfaction, and turnover intention [2,3,8]. Our study, which included nurses from ICUs, oncology units, and EDs, adds evidence that this association persists after adjustment for selected sociodemographic characteristics. Furthermore, the comparison across three high-acuity specialties provides additional insight into variations in moral distress within the Saudi Arabian healthcare context, extending the geographical and clinical scope of existing evidence.

The findings are consistent with Jameton’s conceptualization of moral distress, which proposes that repeated exposure to situations in which nurses are unable to act according to their ethical judgment results in the accumulation of moral residue. Over time, unresolved moral distress may be associated with erosion of professional integrity, increased emotional exhaustion, and greater likelihood of reported withdrawal intention from the workplace. The finding that moral distress remained significantly associated with turnover intention after adjustment for selected sociodemographic characteristics is consistent with this theoretical proposition and suggests that the psychological consequences of unresolved ethical conflict may not depend on the sociodemographic characteristics examined. Furthermore, from the perspective of COR theory, persistent moral distress may be associated with depletion of nurses’ emotional and professional resources, such that withdrawal intention may represent a reported adaptive response when opportunities to restore these resources are perceived as limited.

Practically, these findings suggest that efforts to reduce nurse turnover may need to extend beyond recruitment, financial incentives, or staffing policies alone. Healthcare organizations could consider initiatives that address moral distress, such as accessible ethics consultation services, structured ethical debriefing following morally challenging situations, supportive leadership, and organizational cultures that encourage open discussion of ethical concerns without fear of reprisal. Although these initiatives may offer opportunities to address ethical challenges in clinical practice, their effectiveness in reducing moral distress or improving nurse retention requires evaluation in prospective or interventional studies. Among the sociodemographic characteristics examined, clinical department was associated with both moral distress and turnover intention. Nurses working in ICUs and oncology units reported significantly higher levels of moral distress than those working in EDs. Although a statistically significant overall difference in turnover intention across departments was observed, post hoc pairwise comparisons did not identify significant differences between any individual pair of specialties, indicating that the overall difference could not be attributed to any specific departmental contrast. These findings suggest that the ethical challenges experienced by nurses may vary according to the nature of their clinical practice and patient population.

The higher moral distress observed among ICU and oncology nurses may reflect the complex ethical dilemmas commonly encountered in these settings, including prolonged life-sustaining treatment, end-of-life decision-making, non-beneficial interventions, and conflicts between patients’ best interests and family or organizational expectations. These findings are consistent with previous studies reporting that ICU and oncology nurses frequently experience greater moral distress because of repeated exposure to ethically challenging situations involving critically ill patients and end-of-life care [3,5]. However, the present study extends previous research by directly comparing three high-acuity specialties within the same healthcare system, thereby limiting the organizational and contextual differences that may complicate comparisons across separate studies. The comparatively lower moral distress observed among ED nurses could be related to the more episodic nature of patient care in emergency settings, where rapid clinical decision-making and shorter patient encounters may reduce prolonged exposure to ethically distressing situations, although emergency nurses continue to face distinct ethical challenges associated with time-critical decision-making and resource constraints. However, this explanation is hypothetical and requires empirical investigation.

Female nurses reported significantly higher moral distress than male nurses, whereas turnover intention did not differ according to sex. Although previous studies have reported inconsistent findings regarding sex differences in moral distress, the higher levels observed among female nurses may reflect structural and occupational factors. Nevertheless, the absence of a corresponding sex difference in turnover intention suggests that although female nurses reported greater moral distress, this did not necessarily translate into greater turnover intention.

By contrast, age, years of clinical experience, nationality, and educational level were not significantly associated with either moral distress or turnover intention. These findings suggest that ethical challenges are experienced across diverse demographic groups and that moral distress may be related more closely to the characteristics of the clinical environment than to these demographic attributes. Consequently, organizational efforts to address moral distress may be better directed at ethical work environments than at specific demographic groups.

### 4.1. Study Limitations

This study has several limitations. First, the cross-sectional design limits the ability to infer causal relationships between moral distress and turnover intention. Second, the reliance on self-reported data may introduce response bias. Third, the study was conducted within selected clinical settings, which may limit the generalizability of the findings to other healthcare contexts. Further, the convenience sampling approach may have introduced self-selection bias, as nurses who volunteered to participate may have differed from those who did not. Finally, although several sociodemographic variables were included in the regression model, other potentially relevant factors, such as organizational ethical climate, leadership, burnout, moral resilience, staffing adequacy, and job satisfaction, were not examined and may also be associated with turnover intention.

### 4.2. Implications for Policy and Future Research

The findings of this study highlight the importance of recognizing moral distress as a factor associated with nurses’ turnover intention in high-acuity clinical settings. Healthcare organizations may consider approaches that address moral distress as part of broader workforce retention strategies, including ethics support services, structured ethical debriefing, supportive leadership, and opportunities for nurses to participate in ethical decision-making. At the organisational level, addressing structural contributors to moral distress, including excessive documentation requirements, inadequate staffing and pressures surrounding potentially inappropriate care, may also warrant consideration alongside individual and team-level support. Given the higher levels of moral distress observed among ICU and oncology nurses, targeted interventions tailored to the ethical challenges of these specialties may be particularly beneficial.

Future research should employ longitudinal and multicenter designs to examine the causal relationship between moral distress and actual nurse turnover across different healthcare settings. Further studies should also investigate organizational and psychological factors, such as ethical climate, moral resilience, burnout, and leadership, that may mediate the relationship between moral distress and turnover intention. Additionally, intervention studies evaluating strategies to reduce moral distress should be conducted to identify effective approaches for improving nurse well-being, workforce retention, and the quality of patient care. Finally, future studies using larger, department-stratified samples could explore whether the association between moral distress and turnover intention varies across clinical specialties.

## 5. Conclusions

This study showed that moral distress is a significant correlate of turnover intention among nurses working in ICUs, oncology units, and EDs in Saudi Arabia. This association was positive and remained significant after adjustment for selected sociodemographic characteristics. These findings extend the current Saudi nursing literature by identifying moral distress as an important ethical factor relevant to workforce retention. Addressing moral distress through supportive organizational strategies and ethical work environments may help support nurse well-being and workforce stability.

## Figures and Tables

**Table 1:** Sociodemographic characteristics of participants (N = 160).

Characteristic	N	%	Mean	SD
Age (years)				
	160		33.37	7.23
Years of clinical experience				
	160		8.50	6.11
Sex				
Female	100	62.5		
Male	60	37.5		
Nationality				
Saudi	74	46.3		
Expatriate	86	53.7		
Educational level				
Undergraduate	136	85.0		
Postgraduate	24	15.0		
Department				
ICU	70	43.8		
Oncology unit	52	32.5		
ED	38	23.8		

ICU = Intensive Care Unit; ED = Emergency Department; SD = Standard Deviation.

**Table 2:** Descriptive statistics and internal consistency of the MMD-HP and TIS (N = 160).

Item	MMD-HP Composite Score (F × D)	Frequency	Level of Distress	Cronbach’s α
	Mean (SD)	Mean (SD)	Mean (SD)	
MMD-HP Total Score	128.89 (103.28)			**0.974**
Item 1—Witness healthcare providers giving “false hope”	4.81 (5.22)	1.72 (1.45)	2.14 (1.41)	
Item 2—Follow family’s insistence on aggressive treatment	5.53 (5.19)	2.29 (1.36)	2.13 (1.32)	
Item 3—Feel pressured to conduct unnecessary tests/treatments	5.92 (5.28)	2.16 (1.28)	2.21 (1.37)	
Item 4—Unable to provide optimal care due to cost pressures	4.85 (5.10)	1.93 (1.33)	1.83 (1.33)	
Item 5—Provide aggressive treatment when death is likely	5.11 (4.45)	2.07 (1.24)	2.15 (1.29)	
Item 6—Pressured to avoid reporting medical errors	4.32 (4.54)	1.74 (1.27)	2.02 (1.38)	
Item 7—Care for patients I feel unqualified to care for	4.74 (4.41)	1.86 (1.26)	1.94 (1.41)	
Item 8—Care causing unnecessary suffering	4.38 (4.74)	1.83 (1.34)	1.88 (1.36)	
Item 9—Patient care suffers due to lack of provider continuity	4.98 (5.08)	1.91 (1.33)	2.03 (1.45)	
Item 10—Not discuss prognosis per physician/family request	4.90 (5.03)	1.96 (1.38)	2.08 (1.41)	
Item 11—Witness ethics violation without support for reporting	4.18 (4.87)	1.67 (1.40)	1.76 (1.46)	
Item 12—Participate in care I disagree with due to litigation fears	4.45 (5.17)	1.68 (1.43)	1.81 (1.52)	
Item 13—Work with incompetent healthcare team members	4.63 (4.90)	1.95 (1.33)	1.87 (1.39)	
Item 14—Low-quality care due to poor team communication	4.41 (4.89)	1.64 (1.26)	1.93 (1.47)	
Item 15—Patients not given adequate information for consent	4.49 (5.13)	1.62 (1.39)	1.93 (1.41)	
Item 16—Care for more patients than is safe	5.44 (5.08)	2.03 (1.25)	2.18 (1.34)	
Item 17—Compromised care due to lack of resources	5.04 (4.90)	1.92 (1.32)	2.01 (1.34)	
Item 18—Lack of administrative support for patient care issues	5.09 (4.92)	1.91 (1.28)	2.06 (1.32)	
Item 19—Excessive documentation requirements	6.03 (5.61)	2.16 (1.30)	2.13 (1.43)	
Item 20—Fear retribution if I speak up	4.98 (5.04)	1.91 (1.33)	1.94 (1.35)	
Item 21—Feel unsafe/bullied amongst colleagues	3.98 (4.62)	1.66 (1.36)	1.78 (1.37)	
Item 22—Work with abusive patients/family members	4.93 (4.92)	1.84 (1.26)	2.15 (1.32)	
Item 23—Overemphasize tasks at expense of patient care	4.81 (4.64)	1.90 (1.18)	2.00 (1.25)	
Item 24—Care for patients with unclear treatment plans	4.56 (4.62)	1.82 (1.25)	1.95 (1.35)	
Item 25—Work within power hierarchies compromising care	4.29 (4.75)	1.74 (1.34)	1.82 (1.28)	
Item 26—Team gives inconsistent messages to patient/family	4.73 (4.73)	1.79 (1.28)	1.94 (1.33)	
Item 27—Team members disrespect vulnerable patients	4.35 (4.95)	1.64 (1.42)	1.79 (1.43)	
Overall MMD-HP Total Score	128.89 (103.28)			
TIS Item		Mean	SD	Cronbach’s α
Item 1—I often seriously consider leaving my current job		2.82	1.20	
Item 2—I intend to quit my current job		2.75	1.21	
Item 3—I have started to look for other jobs		2.92	1.47	
TIS Mean Score		2.83	1.19	0.908

MMD-HP = Measure of Moral Distress for Healthcare Professionals; TIS = Turnover Intention Scale; F × D = Frequency × Distress composite score per item, with each item score ranging from 0 to 16 and the total score ranging from 0 to 432; Frequency scores range from 0 (never) to 4 (very frequently); Level of Distress scores range from 0 (none) to 4 (very distressing); TIS items are rated on a 6-point Likert scale ranging from 1 (strongly disagree) to 6 (strongly agree), with higher scores indicating greater turnover intention; SD = Standard Deviation.

**Table 3:** Differences in moral distress and turnover intention by sociodemographic characteristics among nurses (N = 160).

**Characteristic**	**MMD-HP, Pearson’s r**	***p* value**	**TIS, Pearson’s r**	***p* value**
Age (years)	*r* = 0.143	0.074	*r* = 0.030	0.711
Years of clinical experience	*r* = 0.116	0.147	*r* = 0.092	0.245
Sex				
**Characteristic**	**MMD-HP, Mean ± SD**	***p* value**	**TIS, Mean ± SD**	***p* value**
Female	140.16 ± 115.51		2.83 ± 1.28	
Male	109.98 ± 75.84	0.049	2.84 ± 1.04	0.950
Nationality				
Saudi	120.90 ± 85.55		2.93 ± 1.02	
Expatriate	135.75 ± 116.45	0.358	2.75 ± 1.33	0.334
Educational level				
Undergraduate	124.56 ± 100.57		2.81 ± 1.21	
Postgraduate	154.35 ± 117.15	0.260	2.97 ± 1.09	0.504
Department				
ICU	150.68 ± 118.02		2.67 ± 1.25	
Oncology	134.75 ± 96.78	0.003	3.17 ± 1.11	0.047
ED	81.47 ± 61.39		2.68 ± 1.12	

MMD-HP = Measure of Moral Distress for Healthcare Professionals; TIS = Turnover Intention Scale (mean score; range 1–6); *r* = Pearson’s correlation coefficient; ED = Emergency Department; ICU = Intensive Care Unit; SD = Standard Deviation.

**Table 4:** Correlation between moral distress and turnover intention among nurses (N = 160).

Variable	Moral Distress	Turnover Intention
Moral distress	1	
Turnover intention	0.332 *	1

Values are Pearson’s correlation coefficients. * *p* < 0.001.

**Table 5:** Multiple linear regression analysis of factors associated with participants’ turnover intention (N = 160).

Variable	B	SE	β	*t*	*p*	95% CI for B
Constant	2.603	0.563	—	4.627	<0.001	1.492, 3.715
Sex	−0.122	0.240	−0.050	−0.509	0.611	−0.597, 0.353
Nationality	−0.418	0.239	−0.175	−1.751	0.082	−0.890, 0.054
Years of clinical experience	0.023	0.017	0.121	1.404	0.162	−0.010, 0.056
Educational level	−0.201	0.285	−0.060	−0.705	0.482	−0.764, 0.362
Department	0.215	0.118	0.144	1.817	0.071	−0.019, 0.448
Moral distress	0.004	0.001	0.367	4.652	<0.001	0.002, 0.006

Model statistics: R = 0.387; R^2^ = 0.150; Adjusted R^2^ = 0.116; *F*(6, 153) = 4.433; *p* < 0.001. B = unstandardized coefficient; SE = standard error; β = standardized coefficient; CI = confidence interval.

## Data Availability

The raw data supporting the conclusions of this article will be made available by the authors on request.

## Abbreviations

The following abbreviations are used in this manuscript:

ANOVA	Analysis of variance
CHERRIES	Checklist for Reporting Results of Internet E-Surveys
CI	Confidence interval
COR	Conservation of Resources
ED	Emergency department
ICU	Intensive care unit
MMD-HP	Measure of Moral Distress for Healthcare Professionals
SPSS	Statistical Package for the Social Sciences
TIS	Turnover Intention Scale
VIF	Variance inflation factor
